# Supplementary figures and images for: Toxin-Antitoxin Gene Pairs Found in Tn3 Family Transposons Appear To Be an Integral Part of the Transposition Module
Source: mBio. 2020 Mar 31;11(2):e00452-20. doi: 10.1128/mBio.00452-20 (PMC7157771; doi:10.1128/mBio.00452-20)

**A**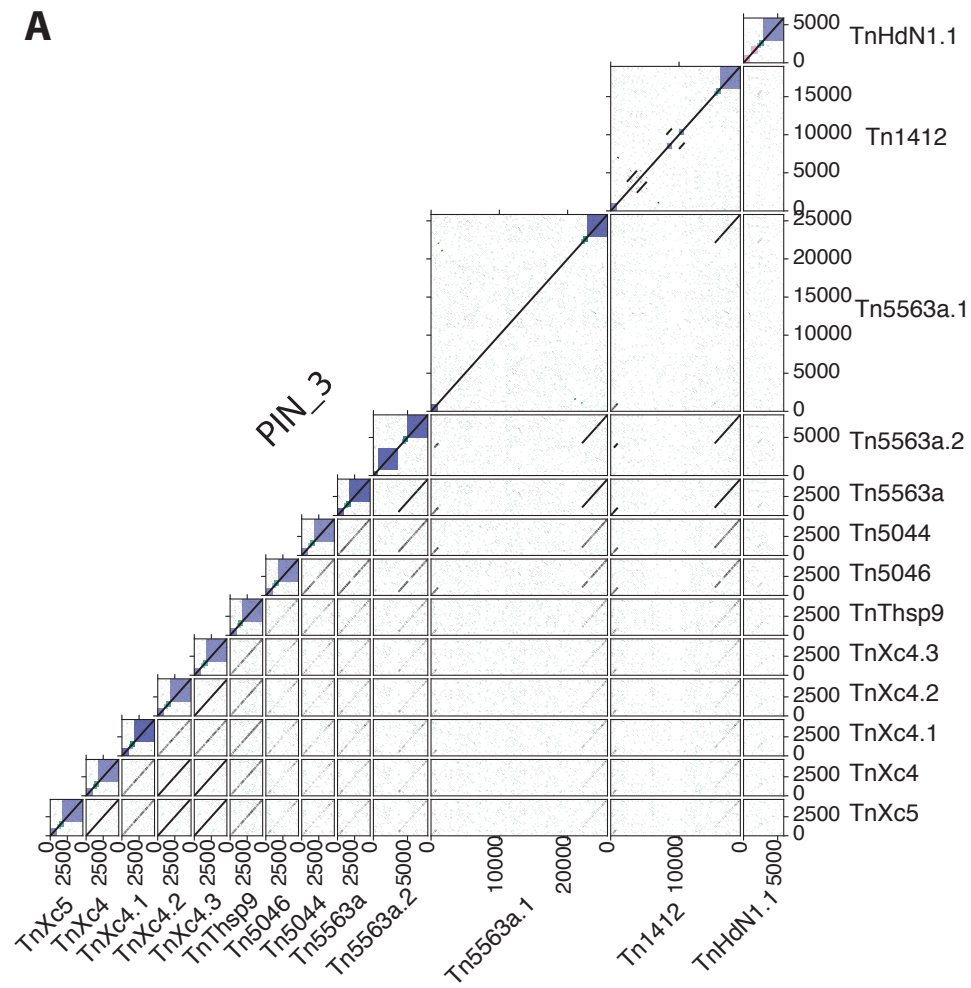**C**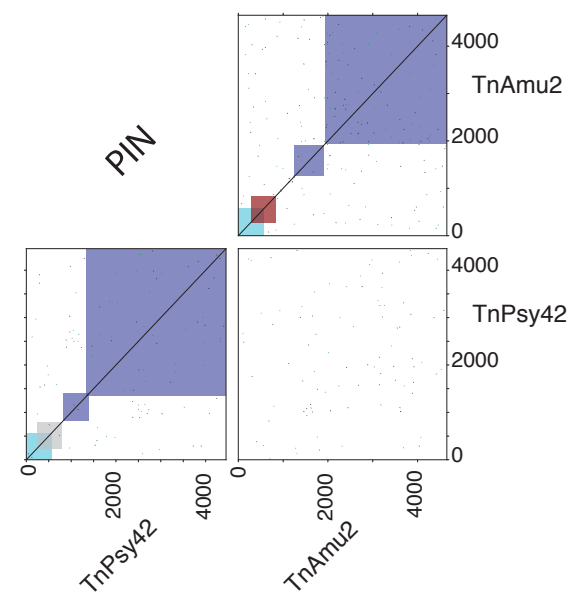**B**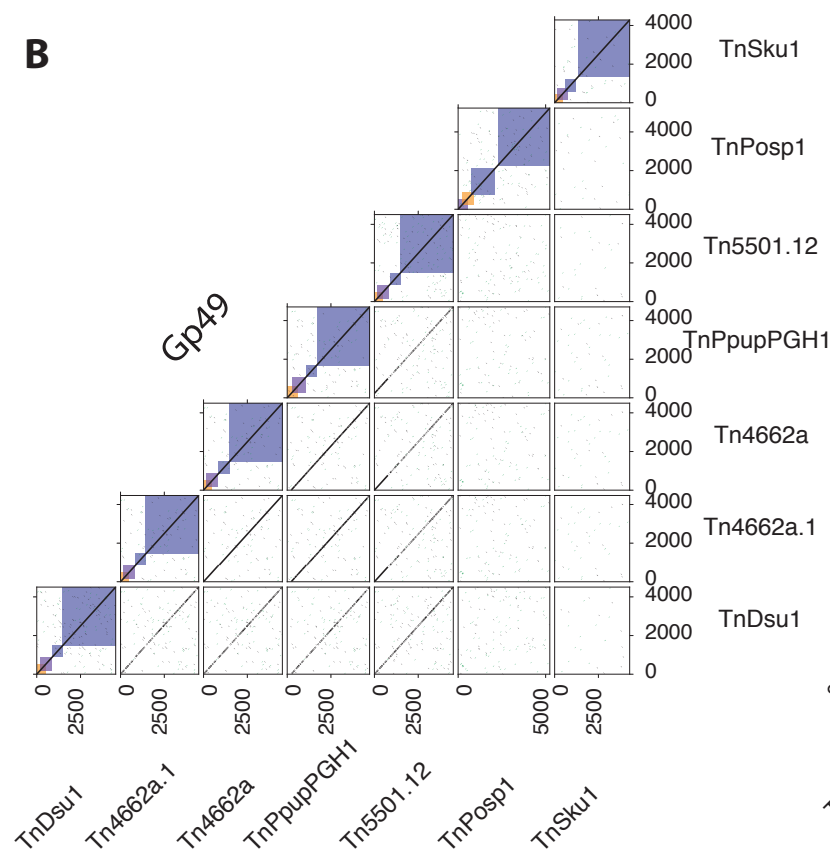**D**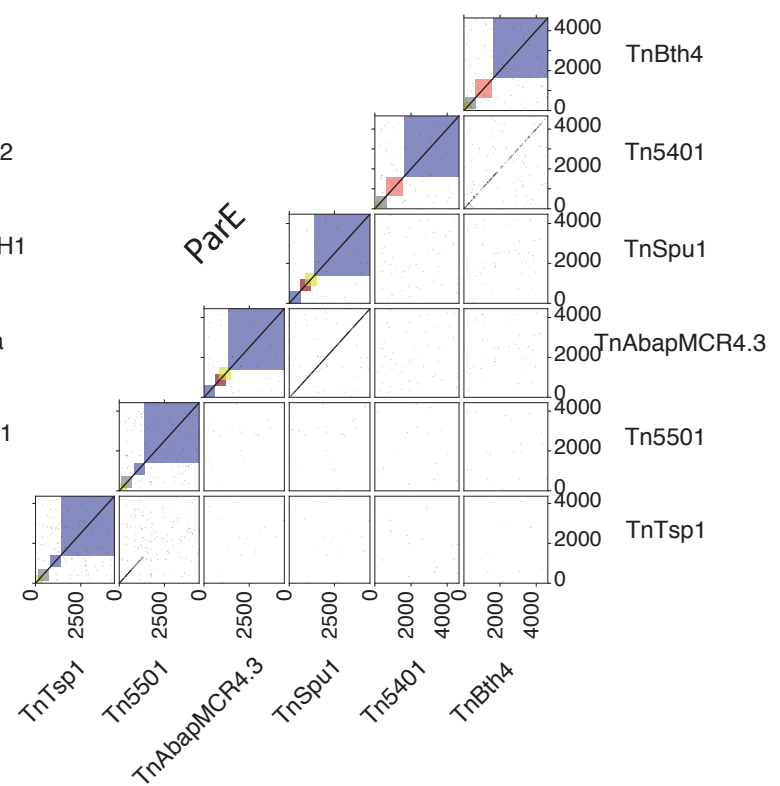

Supplement: FIG S1 [file mBio.00452-20-sf001.pdf]

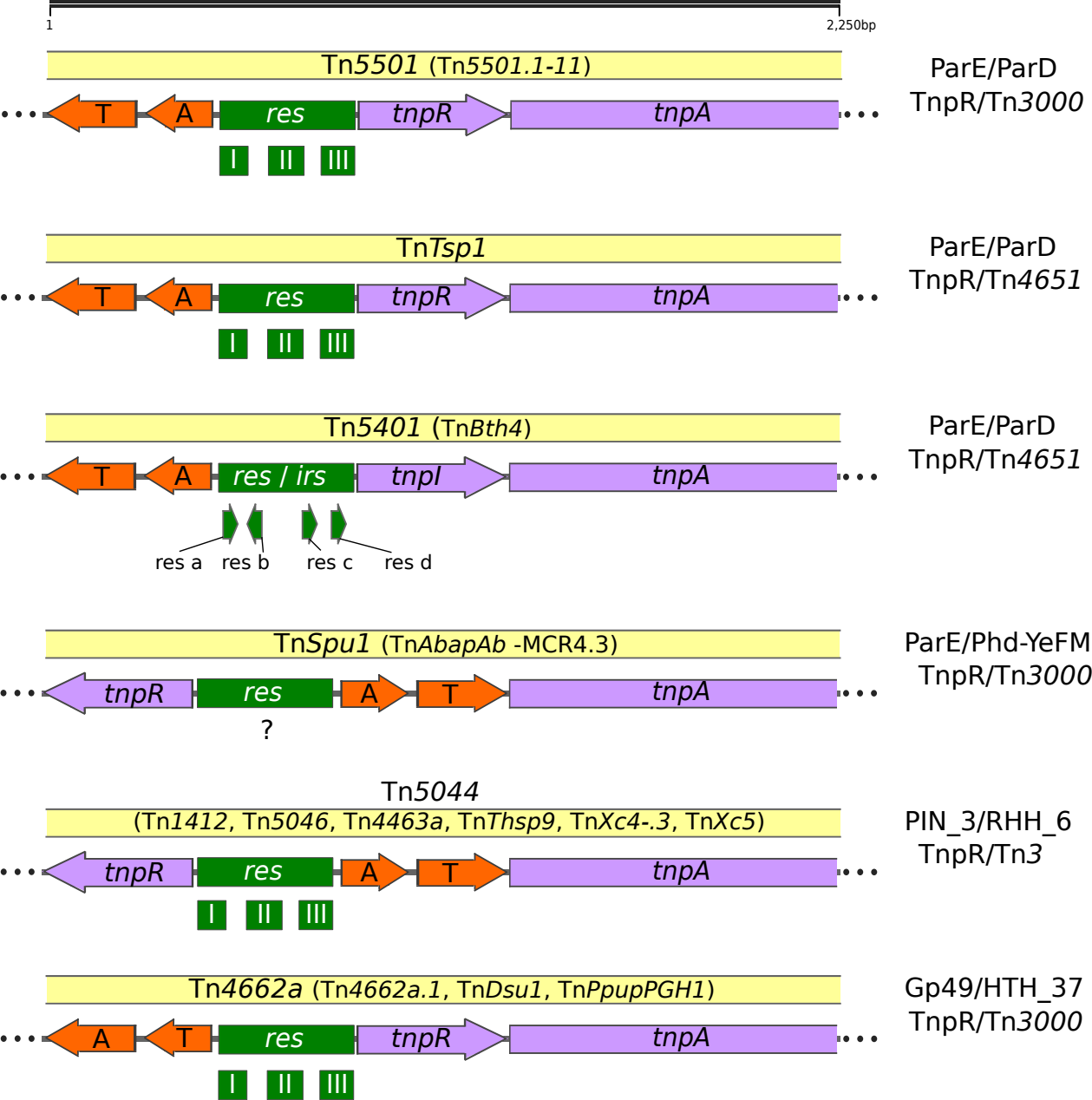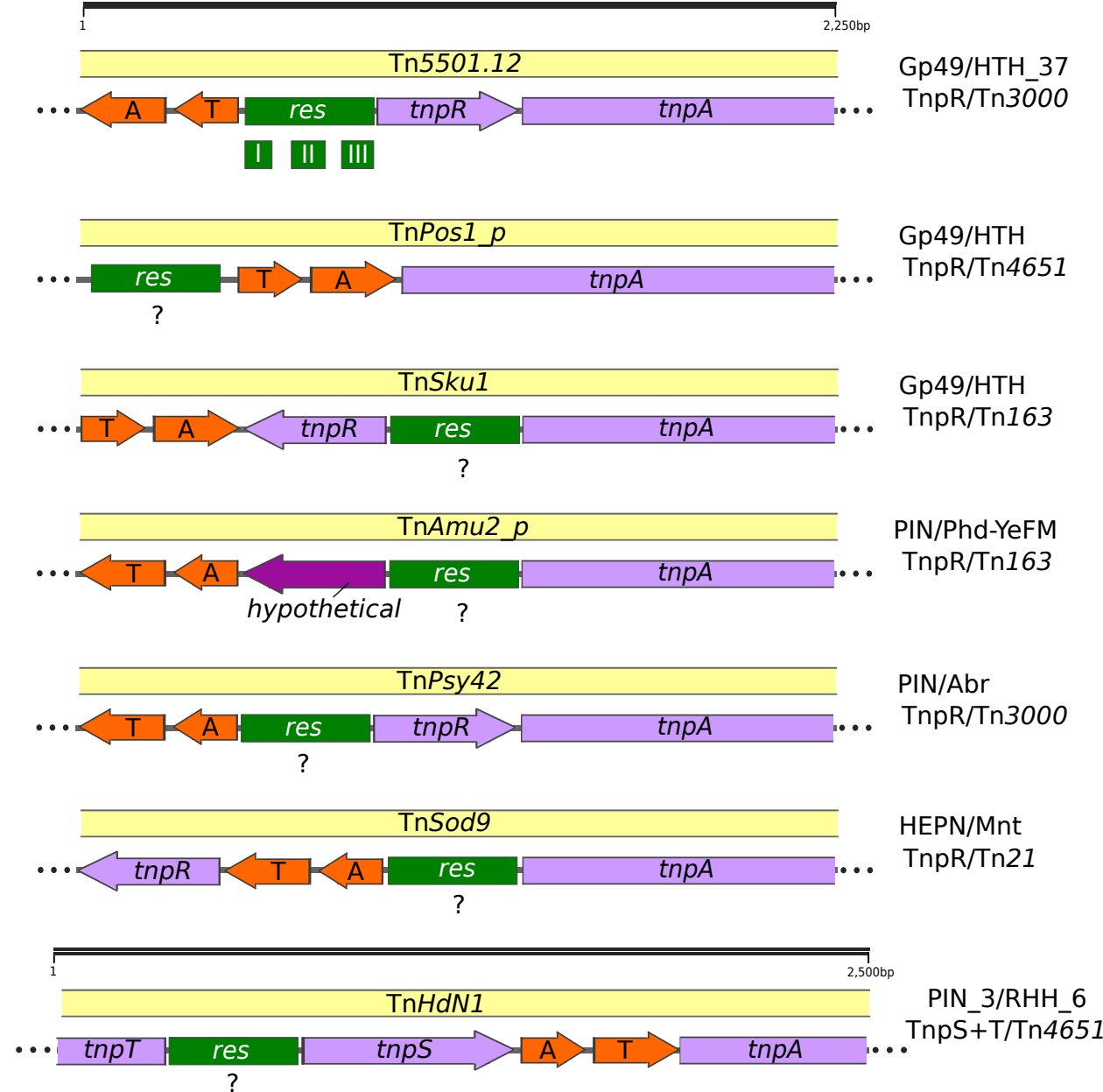

Supplement: FIG S2 [file mBio.00452-20-sf002.pdf]
